# DNA barcoding and molecular identification of field-collected *Culicoides* larvae in the Niayes area of Senegal

**DOI:** 10.1186/s13071-018-3176-y

**Published:** 2018-12-03

**Authors:** Mame Thierno Bakhoum, Mamadou Sarr, Assane Gueye Fall, Karine Huber, Moussa Fall, Mbacké Sembène, Momar Talla Seck, Karien Labuschagne, Laetitia Gardès, Mamadou Ciss, Geoffrey Gimonneau, Jérémy Bouyer, Thierry Baldet, Claire Garros

**Affiliations:** 1 Institut Sénégalais de Recherches Agricoles, Laboratoire National de l’Elevage et de Recherches Vétérinaires, BP 2057 Dakar, Sénégal; 2 CIRAD, UMR ASTRE, F-34398 Montpellier, France; 3 ASTRE, Univ Montpellier, CIRAD, INRA, Montpellier, France; 4 Département de Biologie Animale, Université Cheikh Anta Diop de Dakar, Dakar, Sénégal; 5 Agricultural Research Council-Onderstepoort Veterinary Research, Epidemiology, Parasites and Vectors, Onderstepoort, ZA-0110 South Africa; 6 Department of Zoology and Entomology, University of Pretoria, Pretoria, ZA-0002 South Africa; 7 CIRAD, UMR INTERTRYP, F-34398 Montpellier, France; 8 Centre International de Recherche - Développement sur l’Elevage en zone subhumide, Bobo-Dioulasso 01, BP 454 Burkina Faso; 9 CIRAD, UMR ASTRE, Sainte Clotilde, F- 97491 Réunion, France

**Keywords:** *Culicoides*, Barcoding, Afrotropical, Senegal, African horse sickness

## Abstract

**Background:**

Biting midge species of the genus *Culicoides* Latreille (Diptera: Ceratopogonidae) comprise more than 1300 species distributed worldwide. Several species of *Culicoides* are vectors of various viruses that can affect animals, like the African horse sickness virus (AHSV), known to be endemic in sub-Saharan Africa. The ecological and veterinary interest of *Culicoides* emphasizes the need for rapid and reliable identification of vector species. However, morphology-based identification has limitations and warrants integration of molecular data. DNA barcoding based on the mitochondrial gene cytochrome *c* oxidase subunit 1 (*cox*1) is used as a rapid and authentic tool for species identification in a wide variety of animal taxa across the globe. In this study, our objectives were as follows: (i) establish a reference DNA barcode for Afrotropical *Culicoides* species; (ii) assess the accuracy of *cox*1 in identifying Afrotropical *Culicoides* species; and (iii) test the applicability of DNA barcoding for species identification on a large number of samples of *Culicoides* larvae from the Niayes area of Senegal, West Africa.

**Results:**

A database of 230 *cox*1 sequences belonging to 42 Afrotropical *Culicoides* species was found to be reliable for species-level assignments, which enabled us to identify *cox*1 sequences of *Culicoides* larvae from the Niayes area of Senegal. Of the 933 *cox*1 sequences of *Culicoides* larvae analyzed, 906 were correctly identified by their barcode sequences corresponding to eight species of *Culicoides*. A total of 1131 *cox*1 sequences of adult and larval *Culicoides* were analyzed, and a hierarchical increase in mean divergence was observed according to two taxonomic levels: within species (mean = 1.92%, SE = 0.00), and within genus (mean = 17.82%, SE = 0.00).

**Conclusions:**

Our study proves the efficiency of DNA barcoding for studying *Culicoides* larval diversity in field samples. Such a diagnostic tool offers great opportunities for investigating *Culicoides* immature stages ecology and biology, a prerequisite for the implementation of eco-epidemiological studies to better control AHSV in the Niayes region of Senegal, and more generally in sub-Saharan Africa.

**Electronic supplementary material:**

The online version of this article (10.1186/s13071-018-3176-y) contains supplementary material, which is available to authorized users.

## Background

Biting midge species of the genus *Culicoides* Latreille (Diptera: Ceratopogonidae) comprise more than 1300 species distributed worldwide [1]. Certain *Culicoides* species are the biological vectors of important arboviruses of livestock worldwide, such as the African horse sickness virus (AHSV), bluetongue virus (BTV), epizootic hemorrhagic disease virus (EHDV), equine encephalosis virus (EEV) and Schmallenberg virus (SBV) [2]. African horse sickness virus is an arbovirus of equids that is biologically transmitted by competent vectors of the genus *Culicoides* [3]. This disease is recorded in Africa and Arabian Peninsula and is ranked among the most lethal of viral infections known to affect horses with mortality rates in naive equine populations that can reach 80–90% [3, 4]. Massive AHS epizootic outbreaks occurred in Senegal in 2007 [5, 6]. Knowledge on the ecology of *Culicoides* will be crucial for the development and implementation of appropriate and effective vector control strategies in order to reduce the impact of *Culicoides*-borne diseases. However, a major limitation is that morphology-based methods for *Culicoides* species identification are time-consuming and require taxonomic expertise. Adult morphological identification may involve dissection and microscopical mounting of specimens. Taking into account that subadult stages of the majority of *Culicoides* species still await discovery [7], morphological species identification of *Culicoides* larvae is not possible. Inaccurate *Culicoides* species identification can have significant impacts on control attempts.

Considering these difficulties, it is essential to use complementary and alternative methods to solve taxonomic problems such as the identification of *Culicoides* larvae. Although molecular tools may be expensive and require specialized equipment, they have been useful over the last decade to deepen knowledge in various areas of biology ranging from systematics to ecology [8–13]. Hebert et al. [9] proposed using the mitochondrial gene cytochrome *c* oxidase subunit 1 (*cox*1) as a DNA-based identification system for all animal species, the so-called DNA barcoding approach. DNA barcoding for species-level identification employs a small portion (≈ 658 bp) of the *cox*1 gene to assign a specimen sequence to a voucher species library [9]. This has gained wide acceptance as a supplementary method to resolve taxonomic ambiguities [9, 14]. However, successful DNA barcoding depends on the distinction between intra- and interspecific genetic divergence. The performance of DNA barcoding can vary within the same group of specimens among geographical regions and ecosystems [15]. Species with large effective population sizes can have high intraspecific genetic diversity, which could overlap with interspecific divergence [16]. Furthermore, imperfect taxonomy also could lead to erroneous identifications [17]. Therefore, morphological and molecular identification have both limitations and advantages, but in the absence of a large body of work on morphological identification of the *Culicoides* immature diversity in the Afrotropical region, advances in molecular identification would be a crucial stepping stone.

In the present study, our objectives were: (i) to establish DNA barcode libraries for adult *Culicoides* species collected in different sites in the Afrotropical region [18]; (ii) to assess the accuracy of the *cox*1 gene in identifying of these *Culicoides* species; and (iii) to test the usefulness of DNA barcoding for species identification on a large dataset of *Culicoides* larvae from the Niayes area of Senegal, West Africa. Our study establishes comprehensive DNA barcode libraries for Afrotropical *Culicoides* of interest prior to future taxonomic research such as metabarcoding.

## Results

### Reference DNA sequence analysis

#### Data description and distance summary

Haplotype data analysis detected 170 unique haplotypes in the DNA reference libraries (Table 1). The average nucleotide frequencies for all 42 species were as follows: A (adenine), 28%; T (thymine), 40%; G (guanine), 15.2%; and C (cytosine), 16.8%. The analysis revealed that interspecific Kimura-2-parameter (K2P) genetic divergence ranged between 0.045–0.201 with a mean genetic distance (MGD) of 0.133; intraspecific K2P genetic divergence ranged between 0–0.107 with an average of 0.009 (Table 1).

**Table 1:** Haplotype characteristics and levels of intra- and interspecific diversity of reference DNA sequences

Taxon	*n*	*n*_*hap*_	Intraspecific	H	Interspecific
*C. austeni*	1	1	–	–	0.2
*C. bolitinos*	13	9	0–0.006 (0.001)	0.91	0.075–0.087 (0.077)
*C. brucei*	3	3	0.002–0.018 (0.007)	1	0.134–0.145 (0.141)
*C. candolfii*	2	1	0	0	0.188
*C. distinctipennis*	7	5	0–0.043 (0.012)	0.904	0.124–0.132 (0.128)
*C. dubitatus*	3	2	0–0.002 (0.001)	0.666	0.156–0.159 (0.157)
*C. enderleini*	21	20	0.002–0.05 (0.01)	0.995	0.104–0.12 (0.109)
*C. engubandei*	2	1	0	0	0.148
*C. grahamii*	3	3	0.022–0.031 (0.025)	1	0.166–0.180 (0.171)
*C. gulbenkiani*	3	3	0.004–0.009 (0.006)	1	0.161
*C. imicola*	17	13	0–0.004 (0.002)	0.963	0.101–0.114 (0.107)
*C. isioloensis*	2	2	0.002	1	0.163–0.166 (0.164)
*C. kanagai*	1	1	–	–	0.174
*C. kibatiensis*	1	1	–	–	0.153
*C. kingi*	7	6	0.004–0.009 (0.005)	0.952	0.092–0.097 (0.094)
*C. kwagga*	2	1	0	0	0.099
*C. leucostictus*	3	3	0.004–0.011 (0.007)	1	0.124–0.127 (0.126)
*C. loxodontis*	2	2	0.006	1	0.101–0.109 (0.105)
*C. macintoshi*	3	3	0.004	1	0.153–0.156 (0.155)
*C. magnus*	6	3	0–0.007 (0.002)	0.6	0.134–0.142 (0.136)
*C. milnei*	1	1	–	–	0.169
*C. miombo*	13	4	0–0.002 (0.0005)	0.423	0.114–0.116 (0.114)
*C. moreli*	6	5	0.004–0.006 (0.003)	0.933	0.193–0.199 (0.195)
*C. murphyi*	15	6	0–0.002 (0.0004)	0.79	0.142–0.147 (0.144)
*C. neavei*	2	2	0.099	1	0.161–0.169 (0.165)
*C. nevilli*	9	9	0.011–0.013 (0.012)	1	0.045–0.164 (0.052)
*C. nivosus*	5	4	0–0.064 (0.016)	0.9	0.160–0.164 (0.162)
*C. ovalis*	1	1	–	–	0.175
*C. oxystoma*	14	12	0–0.022 (0.005)	0.978	0.082–0.104 (0.088)
*C. pseudopallidipennis*	13	8	0–0.009 (0.001)	0.935	0.063–0.072 (0.068)
*C. pycnostictus*	2	2	0.009	1	0.153–0.156 (0.154)
*C. ravus*	3	2	0–0.033 (0.011)	0.666	0.132–0.137 (0.135)
*C. schultzei*	2	2	0.018	1	0.087–0.094 (0.09)
*C. similis*	7	4	0–0.107 (0.017)	0.809	0.132–0.164 (0.152)
*Culicoides* sp. #20^a^	6	5	0–0.004 (0.002)	0.933	0.137–0.145 (0.141)
*Culicoides* sp. #22^a^	5	1	0	0	0.101
*Culicoides* sp. #54^a^	1	1	–	–	0.166
*C. subschultzei*	6	6	0.004–0.011 (0.006)	1	0.045–0.049 (0.047)
*C. tororoensis*	2	1	0	0	0.163
*C. tropicalis*	3	2	0–0.013 (0.004)	0.666	0.127
*C. tuttifrutti*	4	3	0–0.011 (0.003)	0.833	0.063–0.075 (0.071)
*C. zuluensis*	8	6	0–0.015 (0.004)	0.928	0.169–0.201 (0.185)

^a^*Culicoides* sp. #20, *Culicoides* sp. #22 and *Culicoides* sp. #54 are putative new species whose status needs still to be clarified in future taxonomic studies [18]

*Abbreviations*: *n* number of *cox*1 sequences, *n*_*hap*_ number of *cox*1 haplotypes; Intraspecific, range of genetic divergence within taxa (mean), *H* haplotype diversity values; Interspecific, range of genetic divergence between taxa (mean)

#### Identification success rates

**Fig. 1:**
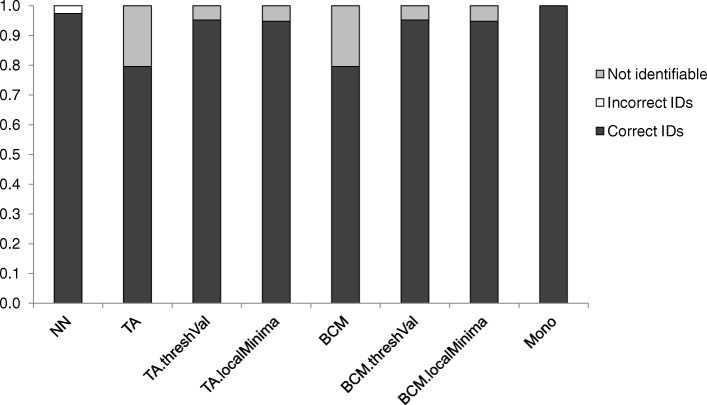
Barplots of measures of identification success. *Abbreviations*: NN, nearest-neighbour; TA, threshold analysis with 1% threshold; TA.threshVal, threshold analysis with 4.4% threshold; TA.localMinima, threshold analysis with 3.59% threshold; BCM, best close match (1% threshold); BCM.threshVal, best close match with 4.4% threshold; BCM.localMinima, threshold analysis with 3.59% threshold; Mono, proportion of monophyly on a NJ tree

In the simulations, the nearest-neighbour (NN) approach returned 97.39% correct and 2.61% incorrect identifications (Fig. 1). The threshold analysis (TA) returned the same results as best close match (BCM) at the threshold value 0.01 (79.56% correct and 20.44% incorrect identifications). With a threshold of 0.039 calculated by the function *localMinima* in SPIDER, the TA and BCM provided 94.68% correct and 5.32% incorrect identifications. With a threshold of 0.044 (Additional file 1: Figure S1) generated by the function *threshVal* in SPIDER, the TA and BCM provided 95.21% correct and 4.79% incorrect identifications. The proportion of monophyly on a neighbor joining (NJ) tree approach (Mono) showed a success rate at 100% (Fig. 1).

#### Barcode gap analysis

In our reference DNA sequences, we counted how often the maximum intraspecific distance exceeded the minimum interspecific distance. Using *length* and *which* functions in SPIDER to query how many times this occurred in our reference DNA sequences, we found that this was the case on 14 occasions (Additional file 2: Figure S2).

### Molecular identification for *Culicoides* larvae

DNA sequences of *Culicoides* larvae collected in the Niayes area of Senegal were successfully obtained for 958 out of 1632 larvae (58.6%). PCR amplifications failed for 99 out of 773 samples of stages L1-L2, while all selected samples of stage L3-L4 were successfully amplified (859/859 samples). This might be explained by the physical size of the different larval stages (L1 and L2 stages are < 2 mm). The sequences were edited in Geneious R11 [19] and 933 *cox*1 sequences of better quality were used in this study. The overall rate of *cox*1 sequences successfully matched within our reference DNA sequences used as Search Set in BLAST search was 97.1%. Thus, 906 out of 933 *cox*1 sequences of larvae were successful identified to *Culicoides* species. However, 27 *cox*1 sequences were unmatched within our DNA barcode reference libraries. In order to find a match, these *cox*1 sequences were used as a query in NCBI (https://blast.ncbi.nlm.nih.gov/Blast.cgi). However, no matches were found for these sequences.

The sequences matched corresponded to eight *Culicoides* species (Table 2). Of these species, *Culicoides oxystoma* Kieffer had the highest percentage (66.8%), followed by *Culicoides nivosus* de Meillon (21.5%), *Culicoides distinctipennis* Austen and *Culicoides similis* Carter, Ingram & Macfie (both slightly above 3%) (Table 2).

**Table 2:** Nucleotide sequence similarity between *Culicoides* larvae sequences and reference partial *cox*1 sequences

*Culicoides* species	Percent similarity Range (Mean)	No. of individuals (% total larvae)
*C. distinctipennis*	98–99 (98.75)	32 (3.5)
*C. enderleini*	97–99 (98.27)	26 (2.9)
*C. imicola*	99	1 (0.1)
*C. kingi*	98–99 (98.94)	17 (1.9)
*C. nivosus*	97–100 (99.01)	195 (21.5)
*C. oxystoma*	97–100 (98.85)	605 (66.8)
*C. pycnostictus*	97	1 (0.1)
*C. similis*	96–99 (98.34)	29 (3.2)

### DNA barcoding database analyses

A total of 1131 *cox*1 sequences were submitted to the BOLD database under the project code “AFCUL” (details see Additional file 3: Table S1). A hierarchical increase in mean divergence was observed according to two taxonomic levels: within species (mean = 1.92%, SE = 0.00) and within genus (mean = 17.82%, SE = 0.00). In the barcode gap analysis using the BOLD Management and Analysis System, situations where the distance to the nearest neighbour was less than the max intra-specific distance were encountered in seven species (Additional file 4: Table S2). Haplotype data analysis detected 360 haplotypes in 1131 *cox*1 sequences for 40 Afrotropical *Culicoides* species.

## Discussion

Our study presents the first DNA barcode analysis of the genus *Culicoides* in the Afrotropical region incorporating adult and larval specimens. Biodiversity questions have become an important issue, not only in the field of conservation but also when species have an economic and health impact such as insects involved in pathogen transmission. *Culicoides*-borne pathogens and notably African horse sickness in the Afrotropical region are of great interest because of major outbreaks affecting horses [2–5, 20, 21]. Moreover, recent studies conducted in west and central Africa revealed high prevalence rates of *Mansonella perstans* both in *Culicoides* specimens and human populations [22–24].

Although of major economic and sanitary importance, the current taxonomic and ecological knowledge of *Culicoides* limits the understanding of the epidemiology of the diseases they transmit and therefore the implementation of appropriate and effective vector control strategies. A major limitation is that morphological methods for identifying *Culicoides* species are tedious and require specialized taxonomic expertise. In addition, species delimitation at the adult stage is complicated by both closely related species, for example the species of the Imicola group [18], and large morphological variations observed within certain species, in particular *C. oxystoma* [18]. Although morphological description and comparison of pupae of certain species has been carried out [25], especially *Culicoides* species related to the Similis group [26] and to the Imicola group [27, 28], there are no morphological identification keys for *Culicoides* larvae.

Generally, two methods have been used to identify *Culicoides* larvae based on the identification of emerging adults: (i) emergence traps covering potential larval habitats and allowing collection and identification of adult midges [29–32]; and (ii) collection of samples from putative breeding sites, such as mud or cattle dung, stored in laboratories for several weeks until adult midges emerge and are identified [27–29, 33, 34]. However, these methods are not suitable for rapid identification due to the potentially lengthy time periods of sub-adults stages, large species diversity and the maintenance efforts required to incubate samples until adult emergence. Indeed, these two methods also have bias in increasing immature mortality and therefore underestimating species diversity. In addition, adult identification problems specific to cryptic species or species with high polymorphism persist.

High-throughput identification of field-collected samples can enable insect vectors monitoring and related eco-epidemiological studies. Species identification using *cox*1 sequence similarity was proposed as a solution to the limitations of morphological taxonomy. The utility of DNA sequences for taxonomic or barcoding purposes is based on the nucleotide divergence [9, 35] and need critical assessment before use. *cox*1 barcoding sequences can be used to discover cryptic species, i.e. closely related and similar morphologically, and, for this reason, overlooked by traditional morphology-based approaches. DNA barcodes can also be used to link different life stages of insects, e.g. larvae, pupae and adults. This is particularly useful in situations where sympatry exists, or larvae are difficult to rear, as frequently occurs for *Culicoides*.

A first attempt to identify *Culicoides* larvae using molecular techniques was conducted by Yanase et al. [36] in a very restricted area in Japan and on a limited number of species. The provision of DNA barcode data for *Culicoides* species, particularly species of medical and veterinary importance in the Afrotropical region, fills an important gap in our knowledge of the phylogeny of these species and identification of immature *Culicoides*. The analysis of the quality of our DNA reference database through distance- and tree-based measures of the identification success rates showed satisfactory results (Fig. 1) and allowed its application to DNA sequences from *Culicoides* larvae collected in various habitats in the Niayes area of Senegal, West Africa, in order to identify species at the larval stage. The abundance of the larval stages for each species needs to be investigated in relation to the type of larval habitat sampled.

Although this study highlights that the barcode database developed here can be reliable for species-level assignments at the larval stage, the possible presence of cryptic diversity within these species is to be taken into account. Our study showed that the most abundant species in the larval sampling was *C. oxystoma*. Considering the vector role of *C. oxystoma* [37–40] its wide distribution (from Africa to South East Asia), previously described ecological heterogeneity and morphological plasticity [8, 41, 42], studies are needed to validate its taxonomic status. *Culicoides oxystoma* might represent a complex of species that require revision.

Of the eight *Culicoides* species identified at the larval stage, *C. imicola* is regarded as the most important vector of African horse sickness [43, 44] and bluetongue viruses [45]; *C. kingi* is involved in the transmission of *Onchocerca gutturosa*, a widespread parasite of cattle in tropical regions [46]; and *C. oxystoma* is a well-known vector of bovine arboviruses such as Akabane virus in Asia [37, 47]. *Culicoides oxystoma* and *C. kingi* are suspected of being vectors of African horse sickness in the Niayes region of Senegal [38, 48] based on their abundance and trophic behaviour. Larvae of *C. oxystoma* occupied several aquatic and semi-aquatic habitats, such as pond edge, lake edge and puddle edge in the Niayes region [33]. Larvae of this species were also found in several aquatic and semi-aquatic habitats in Japan and India, such as paddy fields, stream edges and pond margins [36, 49, 50]. In contrast, the main larval habitat of *C. kingi* in the Niayes region was lake edge [33]. Although adults of *C. imicola* can sometimes be collected in abundance in suction light traps set up at the vicinity of farms or equids in the Niayes region of Senegal [51, 52], only one *cox*1 sequence obtained during this study was identified as *C. imicola*. This confirms our previous observations that *C. imicola* larvae in the Niayes region have specific requirements and probably that favorable breeding sites of *C. imicola* have been poorly sampled or not sampled during our field investigations [33].

## Conclusions

Our study provides a new diagnostic tool to help identify larvae of *Culicoides* at the species level in sub-Saharan Africa. These results are important regarding species of medical and veterinary interest, especially for vectors of AHSV in the Niayes area of Senegal, and serve as a point of reference for future investigations on larval ecology studies and tentative development of larval control measures that need to be selective and environmental-friendly. Besides providing reliable molecular data for species-level assignments of Afrotropical *Culicoides*, our study proves the efficiency of DNA barcode for studying *Culicoides* larval diversity from field samples. Large-scale barcode data for important taxa like *Culicoides* can provide a common platform to researchers from a wide array of biological studies such as taxonomy, ecology, behavior, life histories, vector control and vector-virus relationship. However, it is of prime importance that the name tagged with the generated sequences must be of high accuracy, confirmed with the expertise of a trained taxonomist, to utilize DNA barcode data for routine identification by other biologists [53]. In addition to routine identification, DNA barcode data can also provide insights into further taxonomic research through elucidation of cryptic species and resolving species complexes.

## Methods

### Reference DNA sequences

Reference DNA sequences constituted 230 *cox*1 sequences representing 42 *Culicoides* species (Table 1). These species were collected in different sites in the Afrotropical region [18, 41, 54, 55]. We described summary statistics and analyzed the quality of our reference DNA sequences (230 *cox*1 sequences representing 42 *Culicoides*) by distance- and tree-based measures of identification success rates using R software v.3.3.2 [56] with APE and SPIDER libraries [57, 58]. Every sequence in our reference DNA sequences was considered as unknown and used as a query against the entire data set of identified sequences, and a species name was assigned based on criteria [57]: nearest neighbour (NN), threshold analyses (TA), best close match (BCM), and monophyly of each species (Mono). These criteria are not identification tools, but permit investigation whether sequences can be used for species identification [57, 59]. The barcode gap was calculated and plotted using the maximum intraspecific distance and the minimum interspecific distance. The barcoding gap [17] is an important concept in DNA barcoding. It is assumed that the amount of genetic variation within species is smaller than the extent of variation between species. Genetic distances were calculated using SPIDER employing the Kimura-2-parameter (K2P) distance metric. Haplotype and nucleotide diversity were calculated using DnaSP v.5 [60].

### *Culicoides* larvae sampling

**Fig. 2:**
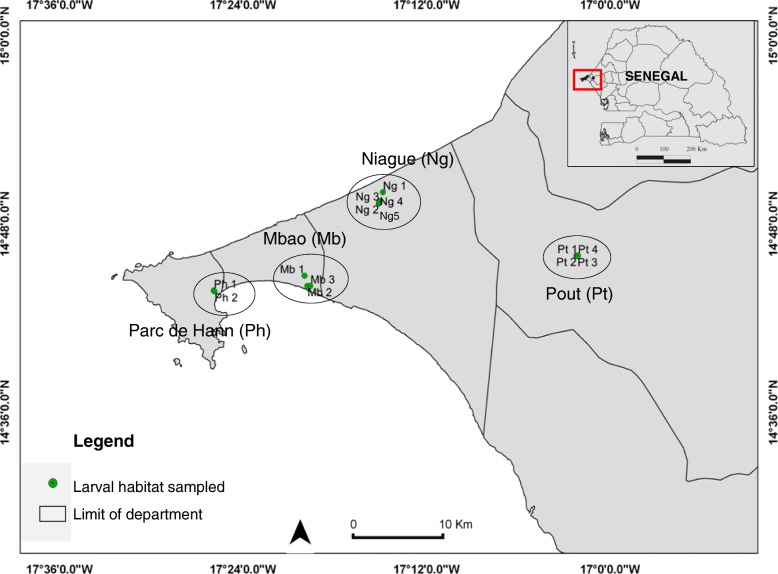
Geographical location of study sites in the Niayes region of Senegal, West Africa. Parc de Hann sites (Ph1 and Ph2) were classified as freshwater lake edge habitats. Mb1, Ng2, Ng3, Ng4 and Ng5 were pond edge while Mb2, Mb3 and Ng1were saltwater lake edge. In Pout, all habitats were puddle edge (Pt1, Pt2, Pt3 and Pt4)

*Culicoides* larvae sampling was performed at four sites in the Niayes region of Senegal, West Africa: Parc de Hann, Mbao, Niague and Pout (Fig. 2). Among these, 14 larval habitats were monitored twice a month from January to December 2015, totaling 24 collection sessions. The 14 larval habitats monitored were characterized as follows: 2 larval habitats of “freshwater lake edge” in Parc de Hann (Ph1 and Ph2), 3 in Mbao (Mb1 of “pond edge”, and Mb2 and Mb3 of “saltwater lake edge”), 5 in Niague (Ng1 of “saltwater lake edge”, and Ng2, Ng3, Ng4 and Ng5 of “pond edge”), and 4 larval habitats of “puddle edge” in Pout (Pt1, Pt2, Pt3 and Pt4) (Fig. 2).

**Fig. 3:**
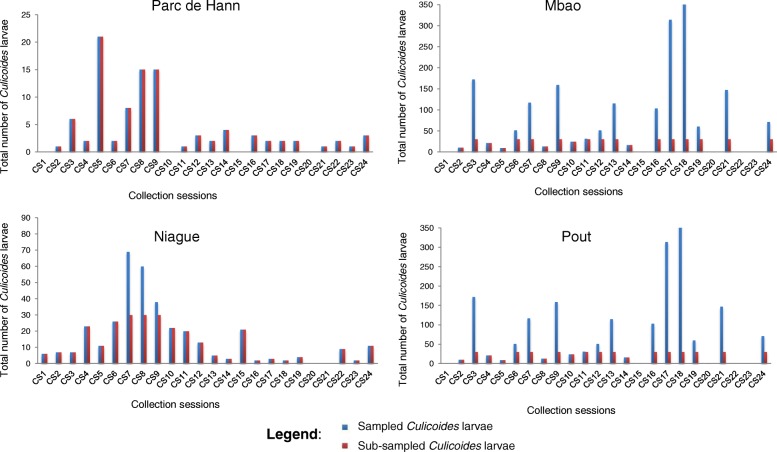
Number of *Culicoides* larvae collected and subsampled per site. A maximum of 30 individuals, irrespective of the numbers collected, were considered for molecular analyses at each of the sites sampled. If less than 30 individuals were collected, all individuals were analyzed

For each habitat, one substrate sample of approximately 650 cm^3^ was collected in the upper layer of the soil surface (0–5 cm) with a trowel, filtered with a fine mesh sieve of 0.8 mm diameter and then investigated for midge larvae using a direct flotation technique in saturated sugar solution (850 g/l). *Culicoides* larvae were collected and preserved in 70% ethanol. A maximum of 30 individuals, irrespective of the numbers collected, were considered for molecular analyses at each of the sites sampled. If fewer than 30 individuals were collected, all individuals were analyzed (Fig. 3).

### DNA extraction, polymerase chain reaction and sequencing

Genomic DNA of larvae *Culicoides* was individually extracted using the NucleoSpin® Tissue DNA Kit (Macherey-Nagel, Duren, Germany) according to the manufacturer’s instructions and maintained at 20 °C until further use. PCR amplification reactions were performed in a 25 μl total reaction volume containing 1× buffer, 1 mM MgCl_2_, 0.2 mM of each dNTP (dATP, dCTP, dGTP and dTTP), 0.2 μM forward primer LCO1490 (5'-GGT CAA CAA ATC ATA AAG ATATTG G-3'), 0.2 μM reverse primer HCO2198 (5'-TAA ACT TCA GGG TGA CCA AAA AAT CA-3') [61], 1.25 U of Taq DNA Polymerase (Qiagen, Hilden, Germany) and 0.4 ng/μl genomic DNA. The PCR cycling conditions were as follows: an initial denaturation step at 94 °C for 5 min followed by 5 cycles of 94 °C for 30 s, 45 °C for 40 s, 72 °C for 1 min, 35 cycles of 94 °C for 30 s, 51 °C for 30 s, 72 °C for 1 min, and a final extension step at 72 °C for 10 min. Positive and negative controls for the amplification reactions were carried out at every PCR round. The PCR products were separated on 1.5% agarose gels and the products were sequenced using the same primers as used in PCR amplifications (https://www.genewiz.com). All generated sequences were deposited in GenBank and BOLD.

### Molecular identification for *Culicoides* larvae

Reference DNA sequences were transformed as a BLAST database using *makeblastdb* of the BLAST software v.2.2.31 [62]. To discriminate *Culicoides* species within the larvae generated sequences, *cox*1 sequences of *Culicoides* larvae were edited in Geneious R11 [19] and used as a query in BLAST search in the BLAST database, considering the different thresholds of divergence generated and used in the identification success rates previously described.

### DNA barcoding database analyses

All DNA sequences in this study (except the sequences of *C. candolfii* Delécolle, Paupy, Rahola & Mathieu [54] (GenBank: KC986403.1 and KC986404.1) and *C. dubitatus* Kremer, Rebholtz-Hirtzel & Delécolle [55] (GenBank: KY707796.1, KY707797.1 and KY707798.1) were submitted to the BOLD database under the project code “AFCUL” for acquiring accession numbers and BOLD-IDs. Sequence alignment was performed using the BOLD Management and Analysis System [63]. Overall data sequences were compared using the *Distance Summary* and *Barcode Gap Analysis* tools on BOLD. In addition, genetic distances were calculated with the BOLD Management and Analysis System, employing the Kimura-2-parameter (K2P) distance metric [64]. Furthermore, haplotypes were calculated using DnaSP v.5 [60].

## Additional files

Additional file 1: **Figure S1.** The minimum cumulative error of false positive and false negative identifications show the optimum threshold; for our DNA reference libraries this was around 4.3 and 4.4%, respectively. (PDF 100 kb)

Additional file 2: **Figure S2.** Line plot of the barcode gap for our DNA reference libraries. For each individual in the dataset, the light sky-blue lines represent the maximum intraspecific distance (bottom of line value), and the minimum interspecific distance (top of line value). The red lines show where this relationship is reversed, and the closest non-conspecific is actually closer to the query than its nearest conspecific, i.e. the situation where there is no barcoding gap. (PDF 3 kb)

Additional file 3: **Table S1.** Details of 1131 *cox*1 sequences representing 40 Afrotropical *Culicoides* species submitted to BOLD database under the project code “AFCUL”. (XLSX 56 kb)

Additional file 4: **Table S2.** Comparison between DNA sequences for Afrotropical *Culicoides* species using the *Barcode Gap Analysis* tools on BOLD. (XLSX 12 kb)

## Abbreviations

AHSV: African horse sickness virus
BCM: best close match
BOLD: Barcoding of Life Database
BTV: Bluetongue virus
EEV: Equine encephalosis virus
EHDV: Epizootic hemorrhagic disease virus
K2P: Kimura 2-parameter
Mono: Monophyly
*cox*1: Mitochondrial gene cytochrome *c* oxidase subunit 1
NJ: Neighbor-joining
NN: Nearest-neighbour
SBV: Schmallenberg virus
TA: Threshold analysis
