# Supplementary figures and images for: DNA barcoding and molecular identification of field-collected Culicoides larvae in the Niayes area of Senegal
Source: Parasit Vectors. 2018 Dec 3;11:615. doi: 10.1186/s13071-018-3176-y (PMC6276223; doi:10.1186/s13071-018-3176-y)

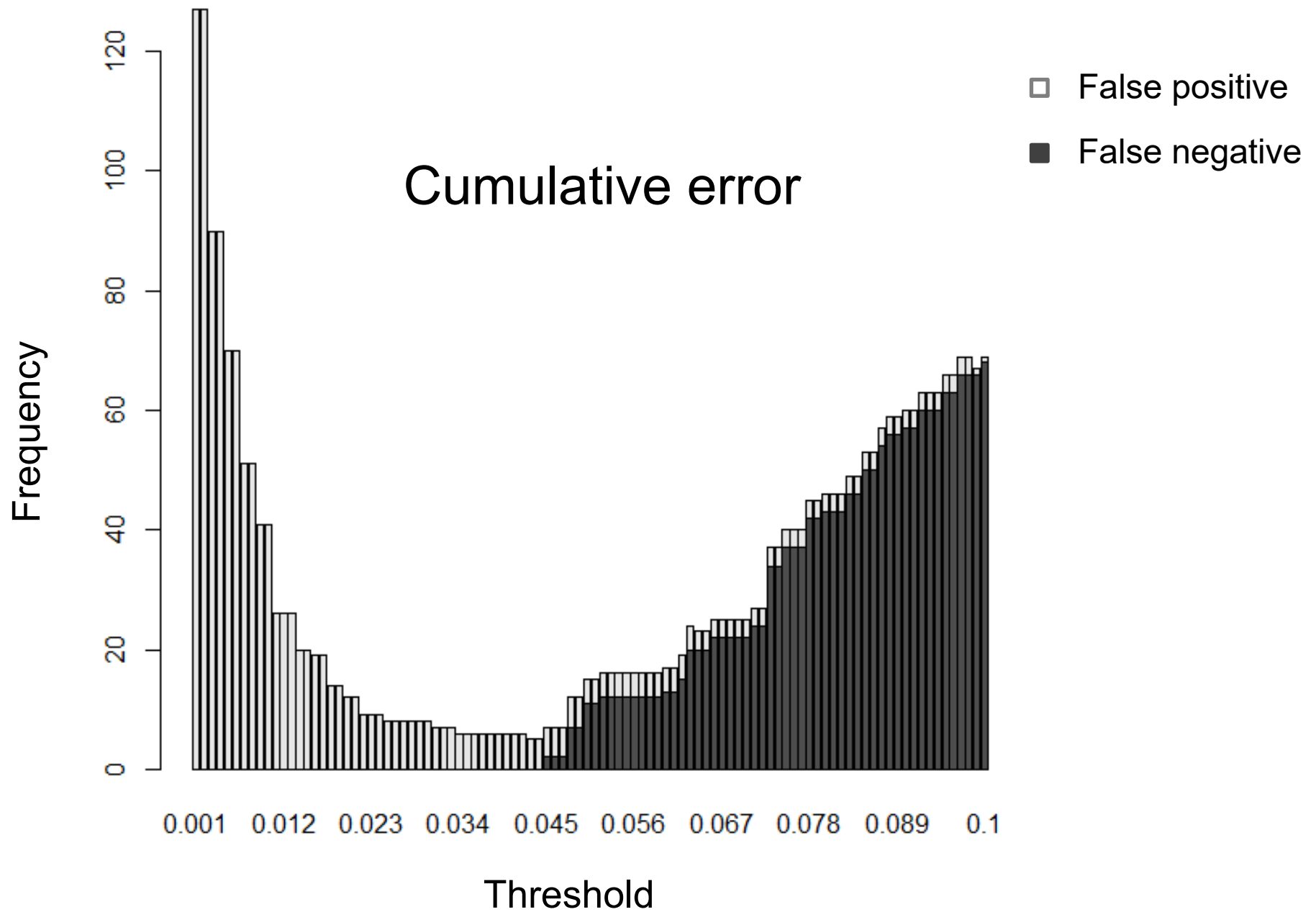

Supplement: Supplementary file 1 — Figure S1. The minimum cumulative error of false positive and false negative identifications show the optimum threshold; for our DNA reference libraries this was around 4.3 and 4.4%, respectively. (PDF 100 kb) [file 13071_2018_3176_MOESM1_ESM.pdf]

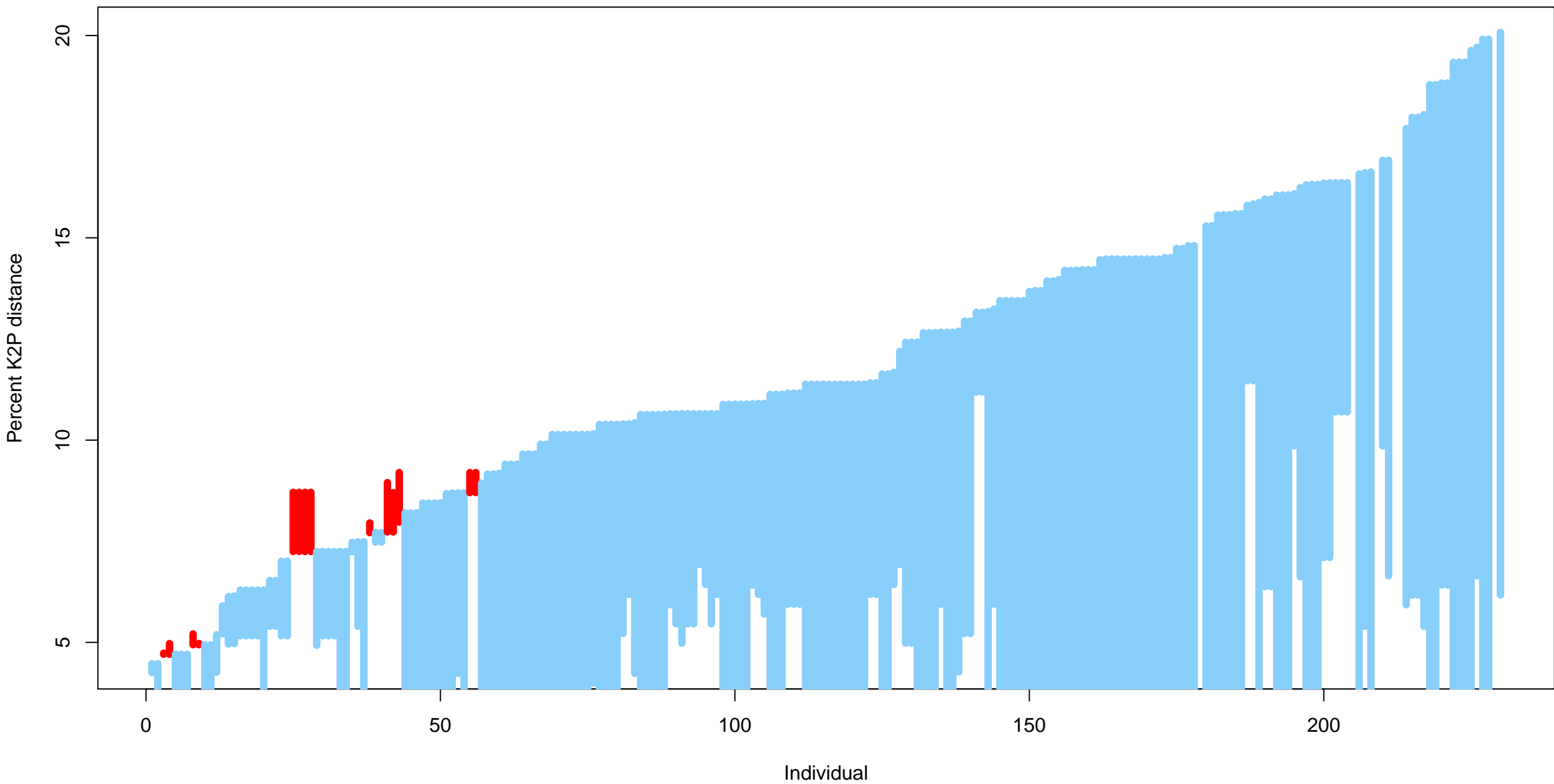

Supplement: Supplementary file 2 — Figure S2. Line plot of the barcode gap for our DNA reference libraries. For each individual in the dataset, the light sky-blue lines represent the maximum intraspecific distance (bottom of line value), and the minimum interspecific distance (top of line value). The red lines show where this relationship is reversed, and the closest non-conspecific is actually closer to the query than its nearest conspecific, i.e. the situation where there is no barcoding gap. (PDF 3 kb) [file 13071_2018_3176_MOESM2_ESM.pdf]
